# Impaired Functionality of Antiviral T Cells in G-CSF Mobilized Stem Cell Donors: Implications for the Selection of CTL Donor

**DOI:** 10.1371/journal.pone.0077925

**Published:** 2013-12-04

**Authors:** Carola E. Bunse, Sylvia Borchers, Pavankumar R. Varanasi, Sabine Tischer, Constança Figueiredo, Stephan Immenschuh, Ulrich Kalinke, Ulrike Köhl, Lilia Goudeva, Britta Maecker-Kolhoff, Arnold Ganser, Rainer Blasczyk, Eva M. Weissinger, Britta Eiz-Vesper

**Affiliations:** 1 Institute for Transfusion Medicine, Hannover Medical School, Hannover, Germany; 2 Department of Hematology, Hemostasis, Oncology and Stem Cell Transplantation, Hannover Medical School, Hannover, Germany; 3 Institute for Experimental Infection Research, TWINCORE, Centre for Experimental and Clinical Infection Research, a joint venture between the Hannover Medical School (MHH) and the Helmholtz Centre for Infection Research (HZI), Hannover, Germany; 4 Institute of Cellular Therapeutics, Hannover Medical School, Hannover, Germany; 5 Department of Pediatric Hematology and Oncology, Hannover Medical School, Hannover, Germany; 6 Integrated Research and Treatment Centre Transplantation, IFB-Tx, Hannover Medical School, Hannover, Germany; INRS - Institut Armand Frappier, Canada

## Abstract

Adoptive transfer of antiviral T cells enhances immune reconstitution and decreases infectious complications after stem cell transplantation. Information on number and function of antiviral T cells in stem cell grafts is scarce. We investigated (1) immunomodulatory effects of G-CSF on antiviral T cells, (2) the influence of apheresis, and (3) the optimal time point to collect antiviral cells.

CMV-, EBV- and ADV-specific T cells were enumerated in 170 G-CSF-mobilized stem cell and 24 non-mobilized platelet donors using 14 HLA-matched multimers. T-cell function was evaluated by IFN-γ ELISpot and granzyme B secretion. Immunophenotyping was performed by multicolor flow cytometry.

G-CSF treatment did not significantly influence frequency of antiviral T cells nor their *in vitro* expansion rate upon antigen restimulation. However, T-cell function was significantly impaired, as expressed by a mean reduction in secretion of IFN-γ (75% *in vivo*, 40% *in vitro*) and granzyme B (32% target-independent, 76% target-dependent) as well as CD107a expression (27%). Clinical follow up data indicate that the first CMV-reactivation in patients and with it the need for T-cell transfer occurs while the donor is still under the influence of G-CSF.

To overcome these limitations, T-cell banking before mobilization or recruitment of third party donors might be an option to optimize T-cell production.

## Introduction

Peripheral blood stem cells (PBSCs), which can be collected after granulocyte colony-stimulating factor (G-CSF) treatment, have been increasingly used for allogeneic stem cell transplantation in recent years [1]. About 80% of all transplantations listed in the European Blood and Marrow Transplant (EBMT) registry in 2008 were PBSC grafts [2]. Benefits of PBSC transplantation are a faster engraftment and less transplant-related mortality, while acute graft-versus-host disease (aGvHD) is comparable with bone marrow transplantation [3], [4], [5].

Apart from GvHD, infectious complications of persistent viruses like cytomegalovirus (CMV), Epstein-Barr virus (EBV) and adenovirus (ADV) remain a problem [6]. Monitoring for viral reactivation and preemptive antiviral treatment have successfully decreased mortality and morbidity [7], [8], but recurrent reactivation of latent viruses remains a clinical problem. The recovery of the patient's adoptive immune response through antiviral cytotoxic T lymphocytes (CTLs) is the key to regaining long-lasting control over these pathogens [9]. Several studies have employed adoptive transfer of antiviral CTLs against CMV [10], [11], [12], [13], EBV [14], [15], [16] and ADV [17] and good treatment outcomes were achieved. The main problem of this approach is the source for virus-reactive T cells, as the patients at highest risk of viral complications are those transplanted from seronegative donors [18]. Some studies suggest that the use of third-party donors might solve this problem [19]. Apart from determining the patient's and donor's serostatus, multimer-based monitoring of antiviral T cells helps to identify high-risk patients in need of adoptive therapies [18], [20], [21]. Furthermore, the number of virus-specific donor T cells transferred with the stem cell graft may play a role in patients with seropositive donors by providing early protection against CMV-reactivation [22]. It has been argued that G-CSF administration alters the gene expression profile and functionality of T lymphocytes and other cells involved in immune responses [23], [24], [25], [26], [27]. For example, granzymes and granulysins in CTLs are down regulated during G-CSF treatment, and genes involved in GvHD and antigen presentation are deregulated during and after G-CSF administration [23]. These effects last about two months [24]. Therefore, leukapheresis for donor leukocyte infusion (DLI) was performed prior to or at least six weeks after G-CSF administration in a gene therapy trial [28].

To further clarify the effects of G-CSF on donor T cells, we addressed the questions of whether (1) multimer monitoring and detection of interferon-gamma (IFN-γ)-secreting cells by enzyme-linked immunospot (ELISpot)-based technique after short-term antigen stimulation are suitable tools to evaluate antiviral CTLs in various samples from stem cell donors, (2) G-CSF mobilization and apheresis influence antiviral T-cell levels, and (3) G-CSF treatment influences the functional activity of CTLs both *in vivo* and *in vitro*. To gain insight into the antiviral T-cell repertoire, we determined the frequencies of CMV-, EBV- and ADV-specific T cells in stem cell and HLA-typed platelet donors using overlapping peptide pools and multimer staining. Furthermore, we analyzed clinical data on recipients' and donors' CMV-serostatus and time of first CMV reactivation.

Here, we analyze the influence of G-CSF stimulation on antiviral T-cells to obtain (1) better understanding of the kinetics of antiviral reconstitution, (2) determine the most effective subpopulation of antiviral T cells, and (3) select an optimal primary transplant or third-party donor for patients requiring antiviral T-cell therapy early after HSCT.

## Materials and Methods

### Study cohort and sampling

Written informed consent was obtained from all donors and patients. Sample collection and analyses were part of an extended monitoring program conducted in the course of routine sampling for clinical follow-up. The study was approved by the Ethics Committee at the Hannover Medical School and is registered as #2906 for patients and as #1142-2011 for healthy donors. Sample material was obtained from 170 PBSC donors between February 2011 and December 2012. Each donor's HLA type, CMV and EBV serostatus were determined. ADV serology was not available. The following samples were collected: whole blood before G-CSF mobilization (WB), whole blood after G-CSF mobilization on the day of apheresis (WBM), blood from the apheresis tubing set (A) and an aliquot from the graft (G). All samples were processed within 48 h, as longer storage yielded no reliable results (Figure S1 in File S1). Storing for >48 h led to exclusion of 2 donors and their samples. The total cohort of 320 samples from 168 donors is summarized in Table 1. In addition samples were collected from 24 healthy platelet donors.

**Table 1:** Study Cohort.

Sample type	Total (n)	Multimer		ELISpot		Both
		yes	no	yes	no	
**WB**	16	9	7	13	3	6
**WBM**	58	58	0	0	58	0
**A**	157	52	105	146	10	41
**G**	89	39	50	84	5	33
**all**	320	158	162	243	76	80

Number of samples analyzed by multimer staining and ELISpot according to sample type, w/o excluded samples (168 donors).

Sample sources: (A) apheresis tubing set, (G) graft, (WB) whole blood, (WBM) mobilized whole blood on day of apheresis.

### G-CSF mobilization and apheresis

G-CSF mobilization was performed by administering 10 µg/kg/day of G-CSF (filgrastim, Amgen, Thousand Oaks, USA) for four consecutive days. Peripheral blood samples were collected via venipuncture into collection tubes containing EDTA as an anticoagulant (Monovette system, Sarstedt, Newton, NC). CD34^+^ counts were determined and apheresis was scheduled accordingly. Apheresis was performed using the Cobe® Spectra Version 4.7 (Terumo BCT, Lakewood, USA).

### Reagents for detection and stimulation of antiviral T cells

All reagents/antigens used in this study are abbreviated as follows: multimer (m), peptide (p) or peptide pool (pp)-virus_viral protein_HLA restriction. For example, the multimer containing the HLA-A*02:01-restricted epitope NLVP of CMV protein pp65 is abbreviated “mCMV_pp65_A02”, and the EBNA1 peptide pool is abbreviated “ppEBV_EBNA1”. A nonsense peptide (mNeg) was used to determine unspecific background of multimer staining. A complete list of antigens/reagents is provided in Table S1 in File S1.

### Multimer staining

100 µl of blood (WB, WBM, A) was stained with HLA-matched multimers (Table S1 in File S1) as described previously [18], [21], [29]. In graft (G) and peripheral blood mononuclear cell (PBMC) samples, 20 µl of cell suspension or 1×10^6^ cells were used. Percentages of specific multimer staining were determined by subtracting the non-specific background value (mNeg). Flow cytometric analysis was performed on an FC500 (Beckman Coulter, Krefeld, Germany) as published previously [29]. An overview on the gating strategy and staining examples are provided in Figure S2 in File S1. Dead cells and debris were excluded by gating on lymphocytes in the FSc/SSc, followed by gating on CD3/CD8-double-positive cells and percentage of multimer-positive cells were then detected in this population. For stability testing the surplus sample material from routine post-HSCT monitoring was stored at room temperature or 4°C until further analysis after 24 h, 48 h and 72 h.

### Isolation and culture of PBMCs

PBMCs were isolated from peripheral blood of platelet donors and from HSC donors before (WB) and on the day of donation (WBM, A). Cells were resuspended in T-cell culture medium (T-CM) consisting of RPMI1640 (Lonza, Vervies, Belgium) supplemented with 10% heat-inactivated human AB serum (C.C.pro, Oberdorla, Germany) plus 50 U/ml IL-2 (PeproTech, Hamburg, Germany) and cultured overnight at a density of 1×10^7^ cells per ml in 24-well plates (Sarstedt). G-CSF and/or antigens were added for the various read-out experiments detailed below.

### Staining for G-CSFR

Flow cytometric analysis of G-CSF receptor (G-CSFR) expression was done on PBMCs (WB, A) using peridinin chlorophyll (PercP)-conjugated anti-CD3, fluorescein isothiocyanate (FITC)-conjugated anti-CD4, allophycocyanin (APC)-conjugated anti-CD8 (all BD Biosciences, Heidelberg, Germany), and R-phycoerythrin (R-PE)-conjugated anti-G-CSFR monoclonal antibodies (mAbs) (BioLegend, San Diego, USA). Flow cytometric analyses were performed using a FACSCanto II flow cytometer (BD Biosciences) and FACSDiva software (V6.1.2). At least 30,000 events in the CD3^+^ lymphocyte gate were analyzed, and the proportion of G-CSFR^+^/CD4^+^ and G-CSFR^+^/CD8^+^ cells was expressed as a percentage of all CD3^+^ lymphocytes analyzed. Upregulation of G-CSFR was three-fold on CD4^+^ T cells and 17-fold on CD8^+^ T cells after *in vivo* G-CSF treatment (data not shown).

### Intracellular staining for IFN-γ to determine the optimal G-CSF concentration *in vitro*

PBMCs from platelet donors were incubated for 7 days with 5 to 50 ng/ml G-CSF (PeproTech) [26], [30]. The optimal concentration was determined by intracellular IFN- γ staining. On day 7, cells were transferred to anti-CD3 mAb-coated (OTK3, eBioscience, San Diego, USA) U-bottom 96-well plates at a density of 2×10^6^ cells per well. Cells were incubated with 10 µg/ml Brefeldin A (Sigma-Aldrich) for 12 hours and stained with CD3-PerCP and IFN-γ-R-PE (Beckmann Coulter). Intracellular staining was performed using the IntraPrep Kit (Beckmann Coulter) according to the manufacturer's instructions. At least 30,000 CD3^+^ cells were acquired in the lymphocyte gate. The proportion of IFN-γ^+^/CD3^+^ cells was analyzed and expressed as a percentage of total CD3^+^ lymphocytes. The IFN-γ concentration in untreated PBMCs (controls) was used as the reference value (100%). At 10 ng/ml G-CSF a reduction of IFN-γ-producing cells to 39% compared to control cells was detected (Figure S3 in File S1). As higher doses did not increase the impairment of IFN-γ production, 10 ng/ml G-CSF was used in further experiments.

### Antigen-specific stimulation after *in vitro* treatment with G-CSF

Baseline frequencies of CMV- and EBV-specific T cells in freshly isolated PBMCs were assessed by multimer staining prior to peptide stimulation. 1×10^7^ cells/ml were stimulated in 24-well plates with 10 µg/ml of the respective single HLA-matched peptides (Table S1 in File S1) with or without 10 ng/ml G-CSF for 7 days (n = 24 donors) for 7 days. Cells were harvested, tested by IFN-γ ELISpot, granzyme B ELISpot and CD107a degranulation assay and stained with the respective HLA-matched multimers for flow cytometric analysis (acquisition of 30,000 CD8^+^ cells). Supernatants were analyzed for granzyme B secretion by ELISA (eBioscience). The percentages of CD8^+^ naïve (N), central memory (CM), effector memory (EM) and terminally differentiated effector memory (TEMRA) T-cell subpopulations were analyzed by additional staining with anti-CD62L-APC-Cy7 and anti-CD45RA-PerCP-Cy5.5 mAbs (both BioLegend).

### IFN-γ ELISpot

Virus-specific IFN-γ-producing T lymphocytes were enumerated by IFN-γ ELISpot assay as described previously [31]. Briefly, 2.5×10^5^ PBMCs (WB, A, platelet donor) or 1.25×10^5^ PBMCs (after 7-day stimulation) were plated in 100 µl T-CM/well and incubated overnight with 10 µg/ml CEF pool (positive control, PANATecs, Tuebingen, Germany), 10 µg/µl peptide, 10 µg/ml peptide pool or without antigen (negative control). Spots were counted using an ImmunoScan Core Analyzer and the results analyzed using ImmunoSpot 5.0 Academic software (both from Cellular Technology Ltd., Bonn, Germany). Means of tests were calculated and expressed as spot-forming units (sfu).

### Granzyme B ELISPOT

In order to determine the influence of G-CSF treatment on cytotoxic activity of antiviral T cells, we performed a granzyme B ELISpot assay as described previously. [32]. Briefly, cells from the HLA-A*02:01-positive T2 cell line were used as target cells and were loaded with pCMV_pp65_A02 and beta-2-microglobulin (Sigma-Aldrich) for 2 hours. T cells from 5 different HLA-A*02:01-positive donors were stimulated for 7 days with pCMV_pp65_A02 and incubated with target cells at an effector to target cell ratio (E∶T) of 2∶1. After 4 hours of incubation, plates were washed and biotinylated anti-granzyme B antibody (Mabtech, Stockholm, Sweden) was added. Granzyme B secretion was detected using streptavidin-alkaline phosphatase (Mabtech) and revealed by 5–13 bromo-4-chloro-3-indolyl phosphate/nitroblue tetrazolium (BCIP/NBT Liquid Substrate, Sigma-Aldrich). Spots were counted as described above. To exclude unspecific cytolytic function of the effector cells, unloaded T2 cells were used as target cells as well. The basal cytolytic activity of effector T cells against the unloaded target cells was subtracted from the specific cytolytic values.

### CD107a degranulation assay

Degranulation of antiviral T cells as a surrogate marker for cytotoxicity [33], [34], [35] was assessed by detecting the expression of CD107a on the cell surface (CMV: n = 5, EBV: n = 11). On day 7, 1×10^6^ peptide- or peptide-pool-stimulated PBMCs were restimulated with pCMV_pp65_A02, pEBV_BZLF1_B08, ppCMV_pp65 and ppEBV_BZLF1, respectively. Phycoerythrin-Cyanin 7 (PCy7)-conjugated anti-CD107a antibody (2.5 µl/1×10^6^ cells, BioLegend) was added and cells were cultured at 37°C, 5% CO_2_. After one hour, Monensin (1∶1000, BioLegend) was added and cells were further incubated for 4 hours before staining with anti-CD3 mAb (PerCP, BD) and anti-CD8 mAb (APC, BD).

### Statistics

Statistical analyses were performed using the Mann-Whitney test or Kruskal-Wallis test followed by Dunn's multiple comparison on GraphPad Prism v5.02 software (GraphPad Software, San Diego, CA). Levels of significance were expressed as p-values (*p<0.05, **p<0.01, ***p<0.001).

## Results

### Study cohort

Healthy G-CSF-mobilized stem donors (n = 170) were included in this study. Serology for CMV was available for 139 donors (positive n = 56, borderline n = 2, negative n = 80, not available n = 31). EBV serology was available for 121 donors (positive n = 104, borderline n = 7, negative n = 9, not available n = 49). Determination of ADV serostatus was not possible, as no fast routine assays for detection of various strains are available. Intermediate analysis showed comparable assay results for mobilized samples (WBM, A, G; Figure S4 in File S1). Thus, samples from the apheresis tubing set (A, n = 157) and graft (G, n = 89) were analyzed in the majority of donors. Table 1 summarizes the different samples analyzed in this study.

### Multimer staining in donor samples

#### Sample type has no impact on specific multimer staining

Results from multimer monitoring of routine post-HSCT samples stayed within in the same range for up to 48 h and storage at 4°C resulted in less variation of the staining results (Figure S1 in File S1, Table S2 in File S1). Thus, samples of 2 donors were excluded from further analyses because their samples had been stored longer than 72 h (Table 1).

**Figure 1:**
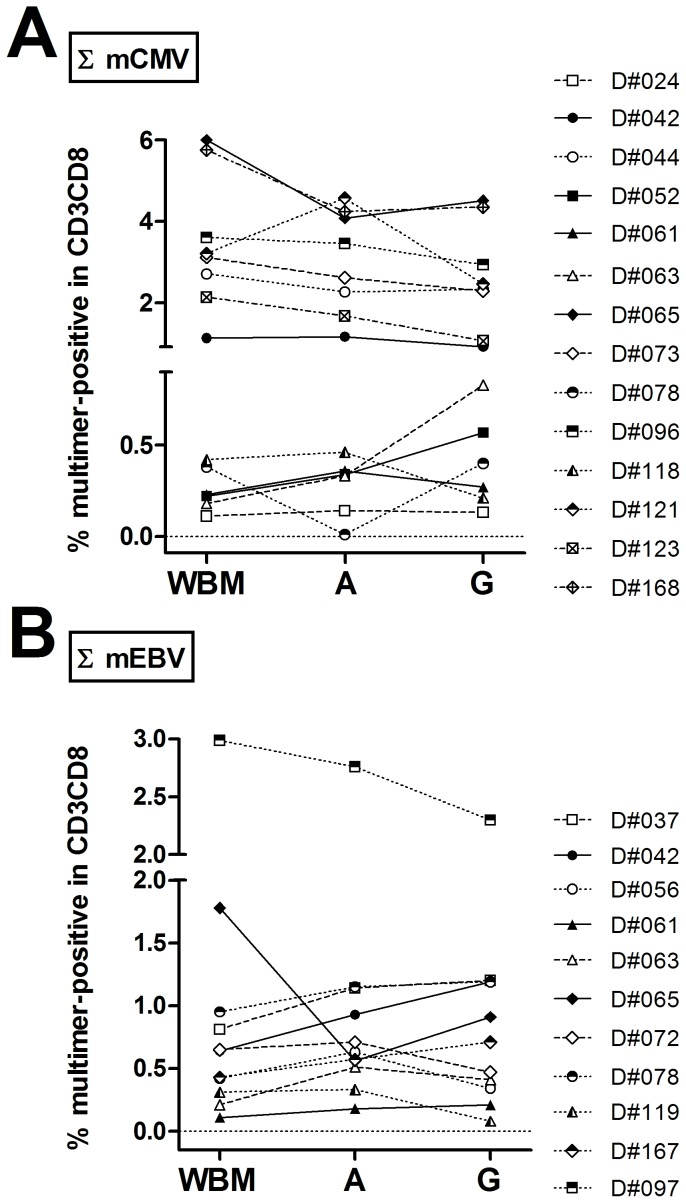
Effects of sample type on multimer staining results. For donors with 3 sample types (WBM, A, G), percentages of multimer-positive cells (sum of all matched alleles) in CD3^+^CD8^+^ T cells were compared for (A) CMV-seropositive (n = 14) and (B) EBV-seropositive (n = 11) donors. Inclusion criteria for the analysis were availability of serostatus and a staining result ≥0.1%. In the majority of samples, the staining results for each sample type remained in the same range. Sample sources: (A) apheresis tubing set, (G) graft, (WBM) mobilized whole blood on day of apheresis.

Non-specific background values (mNeg) differed significantly between the different sample types (Figure S4A in File S1). After background subtraction, overall percentages for specific multimers did not differ significantly by sample type (Figure S4B in File S1). Intra-individual changes were analyzed for mCMV_pp65_A02 and mNeg values for each donor sample set by comparing the values for all CMV-seropositive donors (n = 3) with all four sample types available (Figure S4C–E in File S1). Typical examples of multimer staining in the different sample types and with the different multimers are depicted in Figure S5 in File S1. Comparison of total CMV and EBV CTL values in donors with three types of samples (WBM, A, G) showed that the percentage of multimer-positive cells in CD3^+^CD8^+^ T-cell population remained within the same range (Figure 1A, B; Figure S4 in File S1).

#### Multimer staining in donor samples differentiates seropositive and seronegative individuals

**Figure 2:**
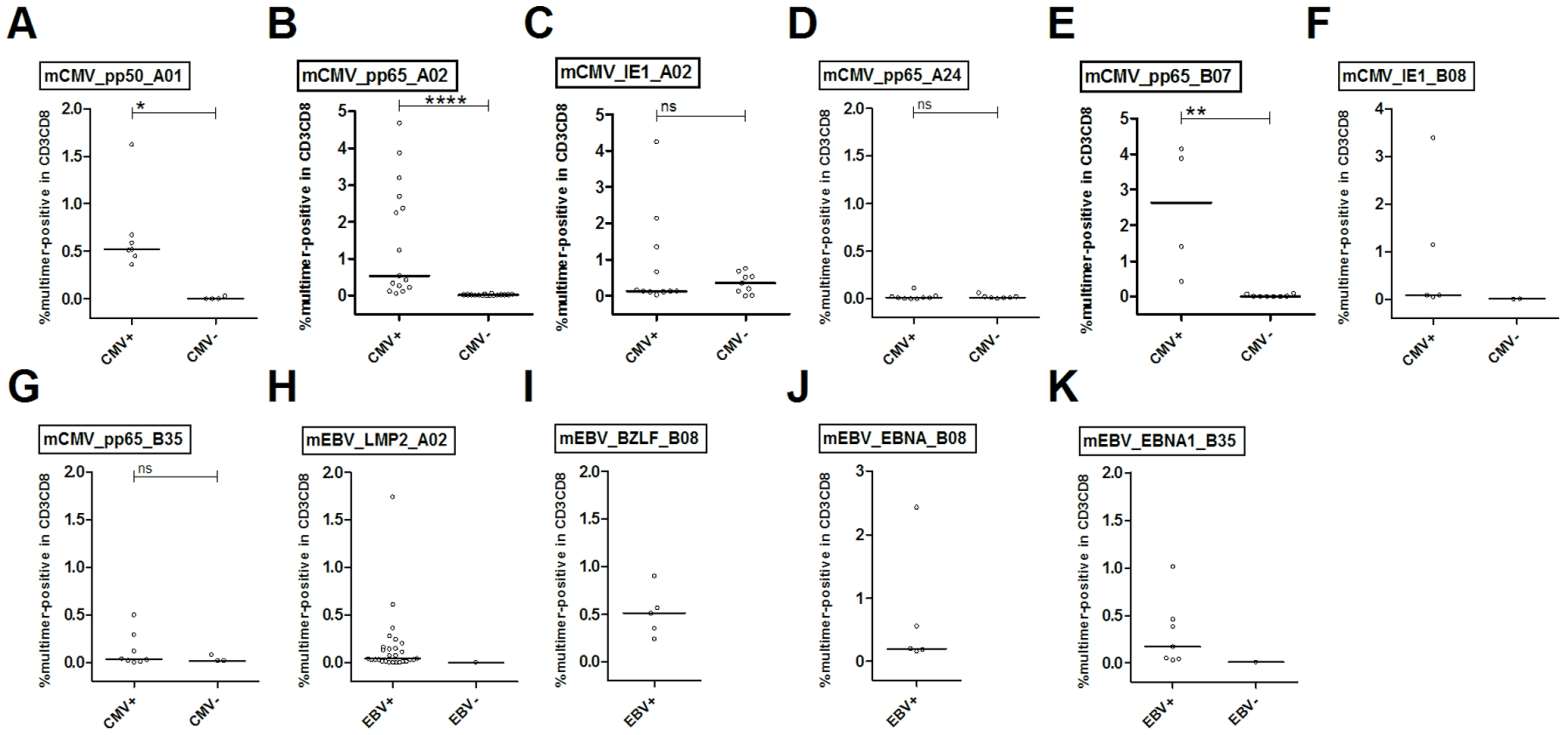
Mean levels of multimer staining analyses in seropositive and seronegative donors. Mean percentages of WB, WBM, A and G samples per donor were calculated for each multimer separately. Donors were grouped according to their CMV and EBV serostatus, respectively, and the resulting groups were analyzed using the Mann-Whitney test. Significant differences between staining results in seropositive and seronegative donors are indicated in the figure and were determined for mCMV_pp50_A01, mCMV_pp65_A02 and mCMV_pp65_B07. For mEBV, no significance differences could be determined due to the small number or complete lack of seronegative donors for the individual multimers. Sample sources: (A) apheresis tubing set, (G) graft, (WB) whole blood, (WBM) mobilized whole blood on day of apheresis.

CMV- and EBV-CTL results were divided into groups based on serostatus. As expected, median levels for mCMV_pp65_A02 (0.53%), mCMV_pp65_B07 (2.46%) and mCMV_pp50_A01 (0.67%) were significantly different between CMV-seropositive and CMV-seronegative individuals (0.02%, 0.02% and 0.01%, respectively) (Figure 2; Table S3 in File S1). A similar analysis could not be performed for EBV, as the number of seronegative patients was too low. For ADV, serotyping was not available.

Surprisingly, the mCMV_IE1-A02 gave false positive staining results in CMV-seronegative individuals (Figure S6 in File S1). This might be due to (i) the higher frequency of naïve precursors for pCMV_IE1_A02-specific CTL. However, detection of naïve precursors in non-enriched material is rather unlikely, as their frequency would be too low [36]. (ii) Furthermore, the VLAELVKQI epitope has some overlap with peptides found in different bacteria and fungi, including anchor amino acids (aa) for HLA-A*02:01 epitopes in position 2 (L) and the auxiliary anchor aa at position 6 (V). Thus, the mCMV_IE1-A02 might display some cross-reactivity (Table S4 in File S1). (iii) The higher avidity of the mCMV_IE1_A02 pentamer might lead to higher background staining compared to the other pentamers used [37].

### Proportion of dominant epitopes for CMV and EBV is not altered in G-CSF mobilized donors compared to healthy blood donors

Recently, we investigated immunodominance of CMV and EBV epitopes in 204 healthy blood and platelet donors and found dominance of pp65 over IE1 and BZLF1 over EBNA-1 and LMP2A [31]. In order to investigate a possible effect of mobilization on the proportions of dominant epitopes, immunodominance of anti-CMV responses was compared for mCMV_pp65_A02 and mCMV_IE1_A02 staining and ELISpot after stimulation of seropositive donors with either ppCMV_pp65 or ppCMV_IE1. Percentages obtained with mCMV_pp65_A02 were higher (Figure 2; median 0.53%), but not significantly different from those obtained with mCMV_IE1_A02 (Figure 2; median 0.13%) in 6 of 11 donors (23/31 samples). In ELISpot assays ppCMV_pp65 led to higher spot counts in 36 of 51 donors (57/72 samples).

Immunodominance of anti-EBV responses was evaluated for mEBV_EBNA3A_B08 and mEBV_BZLF1_B08 staining and ELISpot after stimulation with ppEBV_BZLF1, ppEBV_EBNA1 and ppEBV_LMP2A, respectively. In 16 samples from 5 donors stained with both mEBV_BZLF1_B08 and mEBV_EBNA3A_B08, the BZLF1-derived epitope yielded higher percentages in 3 donors (11 samples; median 0.51% vs. 0.20%; ns). In ELISpot analysis, ppBZLF1 yielded the highest spot counts in 32 of 101 donors (53/156 samples).

The current study results for dominance of CMV- and EBV-specific epitopes from pp65 and BZLF1, respectively, are in concordance with those from our recent study [31]. We found no evidence that G-CSF influences the proportions of epitope-specific antiviral T cells.

### T cells from G-CSF-mobilized stem cell donors secrete less IFN-γ than those from untreated donors

**Figure 3:**
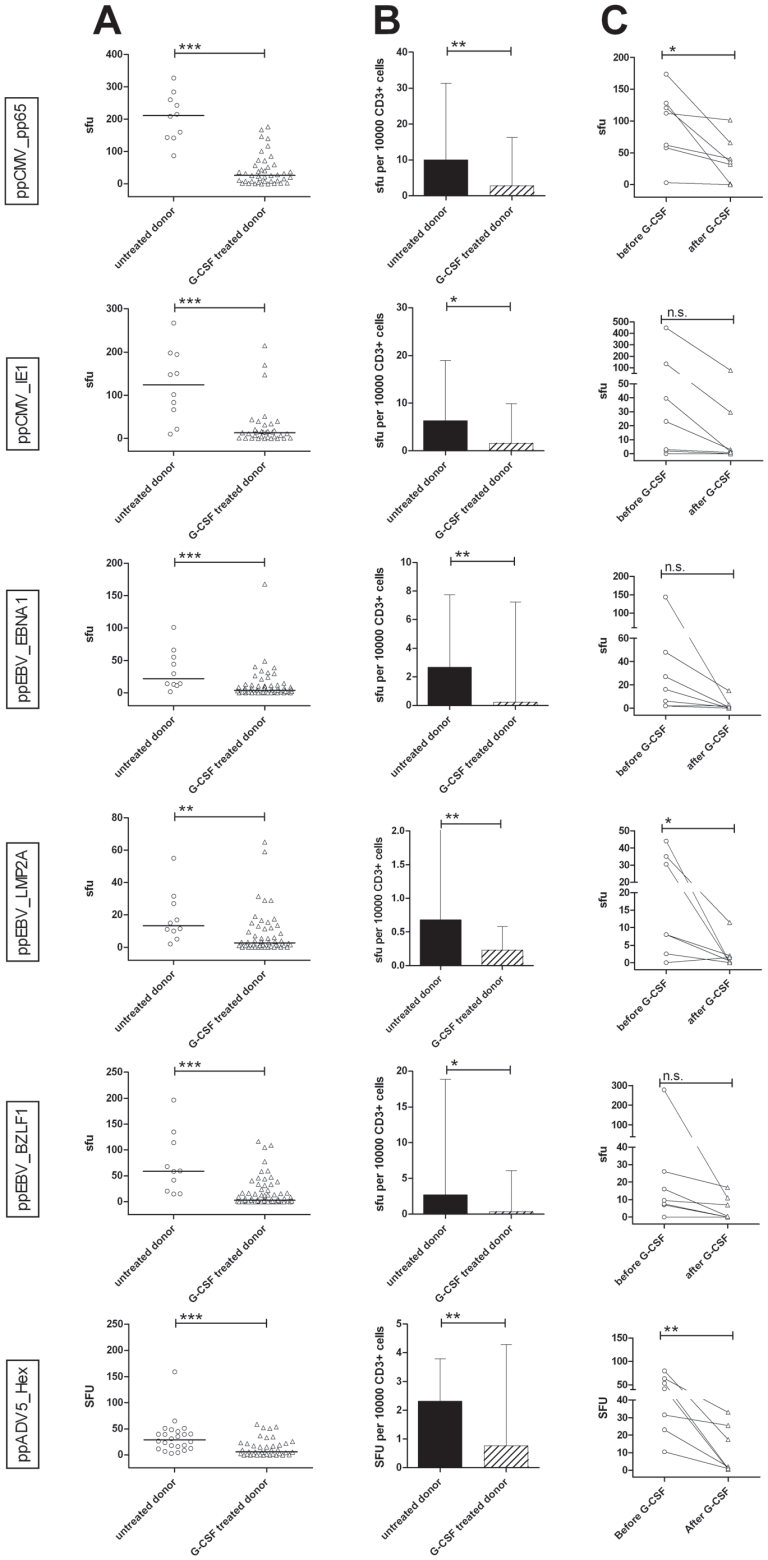
*In vivo* treatment of stem cell donors affects antiviral response to peptide pools for CMV, EBV and ADV. (A) Number of spot forming units (sfu) per well for untreated platelet and G-CSF-treated stem cell donors. (B) Number of sfu per 10,000 CD3^+^ cells for untreated and treated donors. (C) Response of pairs of stem cell donor cells to peptide pools of CMV, EBV and ADV before and after mobilization. Asterisks indicate significant differences (*p<0.05, **p<0.01, ***p<0.001). n.s. = not significant.

T cells from untreated platelet donors (n = 24) and from i*n vivo* G-CSF–mobilized stem cell donors (n = 81) were compared for their IFN-γ production upon stimulation with CMV-, EBV- and ADV-overlapping peptide pools. Analysis of the total number of sfu showed that G-CSF treatment led to a significant reduction in spots for all antigens tested (ppCMV_pp65: median untreated 211.50 sfu, median G-CSF-treated 26.0 sfu; ppCMV_IE1: 124.3 vs. 13.3 sfu; ppEBV_EBNA1: 21.8 vs. 3.8. sfu; ppEBV_LMP2A: 13.3 vs. 2.8 sfu; ppEBV_BZLF1: 59.0 vs. 3.0 sfu; ppADV5_hexon: 28.8 vs. 5.8 sfu) (Figure 3A). This could be confirmed by analysis of sfu/10^4^ CD3^+^ T cells (ppCMV_pp65: 10.09 vs. 2.86, ppCMV_IE1: 6.4 vs. 1.6, ppEBV_EBNA1: 2.7 vs. 0.2, ppEBV_LMP2A: 0.7 vs. 0.2, ppEBV_BZLF1: 2.8 vs. 0.3, and ppADV5_hexon: 2.3 vs. 0.8) (Figure 3B).

Secretion of IFN-γ by cells from the same donor before and after G-CSF treatment (n = 7) was additionally compared (Figure 3C). Again, each donor exhibited a strong reduction of IFN-γ secretion after G-CSF treatment, regardless of the peptide pool used for stimulation.

### *In vitro* G-CSF application reduces T-cell reactivity in response to viral peptides

**Figure 4:**
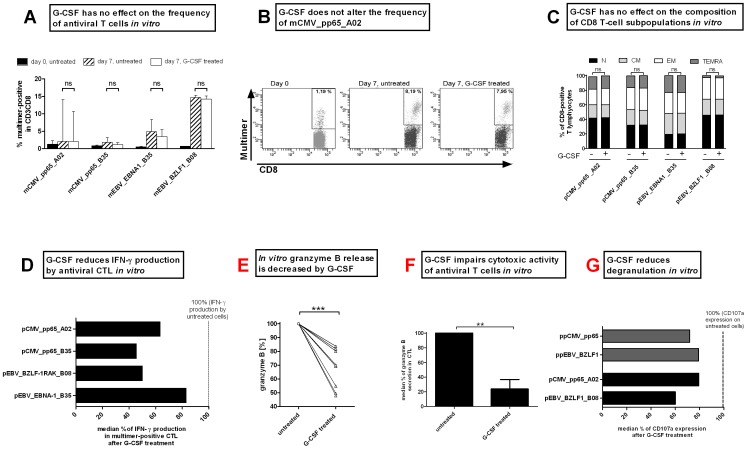
*In vitro* stimulation with G-CSF does not affect the frequency of antiviral T cells but impairs their functionality. (A) The frequency of antiviral T cells was measured by HLA-matched multimer staining before (black bars) and 7 days after G-CSF stimulation (untreated: striped bars and G-CSF-treated: white). Medians with range of independent experiments are displayed. (B) Frequency of mCMV_pp65_A02 in a representative CMV-positive donor on day 0 (before stimulation) and after 7 days of pCMV_pp65_A02 stimulation for untreated and G-CSF treated cells. (C) G-CSF treatment did not change the composition of subpopulation within the of CD8^+^ T-cell population on day 7. (D) IFN-γ secretion as determined by ELISpot analysis after 7 days of peptide stimulation with and without 10 ng/ml G-CSF. The number of IFN-γ secreting units of untreated cells was set to 100%. The number of spot-forming units (sfu) in G-CSF-treated cells is expressed in relation to that in the untreated cells. (E) Granzyme B ELISA, as shown exemplarily for pCMV_pp65_A02 stimulated cells, reveals a reduced secretion of granzyme B after G-CSF treatment. The concentration of secreted granzyme B of untreated cells was set to 100%. (F) G-CSF reduces the cytotoxic activity of antiviral T cells prestimulated over 7 days as shown by granzyme B ELISpot using the pCMV_pp65_A02 loaded T2 cells as target cells. (G) Impaired cytolytic functionality of CD8^+^ T cells was determined by expression of CD107a on the cell surface. The expression of untreated cells was set to 100%.

The frequency, function and immunophenotypes of antiviral T cells from healthy platelet donors treated *in vitro* with 10 ng/ml G-CSF for one week were evaluated by multimer staining, IFN-γ ELISpot, granzyme B ELISpot, granzyme B ELISA and CD107a degranulation assay. The frequency of multimer-positive T cells obtained after 7 days of peptide stimulation did not differ significantly between G-CSF-treated and untreated cells for the single HLA-restricted peptides used (Figure 4A–B; mCMV_pp65_A02: 3.9% vs. 3.4%; mCMV_pp65_B35: 1.7% vs. 1.2%; mEBV_EBNA1_B35: 4.8% vs 3.4%; and mEBV_BZLF1_B08: 10.5% vs 10.4%). The influence of G-CSF on subpopulations of CD8^+^ T cells was additionally tested. No change in the composition of naïve (N), central memory (CM), effector memory (EM) or terminally differentiated effector memory (TEMRA) CD8^+^ T cells was observed after peptide-specific stimulation in the presence or absence of G-CSF (Figure 4C).

T-cell function was evaluated by IFN-γ ELISpot on day 7 (Figure 4D, Figure S7A in File S1); shown is the percentage of sfu/1000 multimer^+^ cells after G-CSF treatment relative to that in untreated multimer^+^ cells (100%). While the frequency of multimer^+^ T cells remained stable, the percentage of IFN-γ secreting cells decreased after G-CSF treatment for pCMVpp65_A02 (63.7%) and pCMVpp65_B35 (45.7%). For EBV, the percentage of IFN-γ producing cells decreased to 83.1% (pEBV_EBNA1_B35) and 50.1% (pEBV_BZLF1_B08).

The immunosuppressive effects of G-CSF treatment on the cytotoxic function of T cells were further assessed by detection of granzyme B secretion and expression of lysosome-associated membrane protein CD107a on the cell surface. Granzyme B was detected .in a target-independent (ELISA, Figure 4E) as well as in a target-dependent assay (ELISpot, Figure 4F, Figure S7B in File S1). In both assays for all donors, a significant decrease in granzyme B secretion was detected (16.31% to 52.19% target-independent, 76.04% target-dependent) after G-CSF-treatment. Further evidence of functional impairment of cytotoxicity after G-CSF treatment was provided by the decreased levels of CD107a on the cell surface (Figure 4G: ppCMV_pp65 71.81%, ppEBV_BZLF1 79.29%, pCMV_pp65_A02 79.59%, pEBV_BZLF1_B08 59.98% of untreated cells; Figure S7C in File S1).

### Relevance of impaired T-cell function in the clinical setting

Follow-up data for seropositive family donors and patients (3/5) showed reconstitution of CMV-CTLs after HSCT, as expected (Figure S8 in File S1). In patient UPN2145 (Figure S8C in File S1), the number of CMV-CTLs expanded upon CMV reactivation on days +41 and +118.

Next, we analyzed the clinical data on the CMV serostatus of recipients (R) and donors (D) and the time of first CMV reactivation in order to determine the demand for adoptive therapies. The proportion of high-risk patients with an R^+^D^−^ CMV-serostatus combination remains at approximately one third of all HSCTs over the last 10 years (Figure S9 in File S1). Here, the seronegative stem cell donors do not qualify as T-cell donors for adoptive antiviral therapy.

Early CMV reactivation (<day +100) occurred in 172 R^+^D^+^ and 103 R^+^D^−^ patients between June 1998 and June 2012. The mean day of CMV reactivation was day +39 and +40 in R^+^D^+^ and R^+^D^−^ patients, respectively. PBSC transplanted from G-CSF treated donors may still be impaired in their function at this early time of CMV-reactivation [23].

## Discussion

Hematopoietic stem cell transplantation is used to cure many malignant, benign and genetic bone marrow disorders, solid tumors, immunodeficiencies, metabolic diseases and autoimmune disorders [38]. G-CSF–mobilized PBSCs have become the preferred source of hematopoietic stem cells for matched related allogeneic HSCT [39], [40].

It is well known that despite to a one-log increase in the number of T cells transplanted with G-CSF–mobilized PBSCs compared to bone marrow grafts no higher rates of severe aGvHD are associated whereas the incidence of chronic GvHD increases [4], [40], [41], [42], [43], [44]. Compared to bone marrow, HLA-identical transplantation of G-CSF–mobilized PBSCs is associated with sustained protection against relapse and that these benefits are not outweighed by GvHD-related mortality [40]. In order to obtain more insight into the role of G-CSF in the modulation of adoptive and innate immune responses, the effect of G-CSF on the functionality of T lymphocytes was investigated in this study.

### Effects of G-CSF on antiviral T cells

G-CSF influences T cells by various mechanisms: (A) It inhibits the secretion of type I cytokines on the single-cell level as well as by reducing the population of cytokine-secreting cells [45], [46], [47]. (B) It induces the polarization of T-cell responses towards Th2 differentiation while inhibiting Th1 proliferation [48], [49]. (C) G-CSF promotes regulatory T cells that produce suppressor cytokines IL-10 and TGF-β [25], [27], [48], [50], and (D) reduces the number of Th17 cells approximately 3-fold [23]. Little is known so far about its effects on antigen-specific CD8+ T cells, one of the main defense mechanisms against viruses. G-CSF acts through its receptor, G-CSFR, which is expressed on myeloid progenitors, granulocytes, monocytes/macrophages, endothelial cells, B cells and activated T cells [51]. In our study, an increase in G-CSFR expression was observed on *in vivo* G-CSF-mobilized T–cell populations suggesting that this cytokine has a direct effect on T cells.

By evaluating the effector function of antiviral T cells after *in vitro* and *in vivo* G-CSF treatment, we were able to show that G-CSF mobilization does not affect the frequency of antiviral T lymphocytes, but strongly impairs the functionality by reducing the capability to secrete IFN-γ (40% to 75% reduction) and cytolytic molecules like granzyme B (32% reduction). The reduced cytolytic function as determined by the reduction of granzyme B release in a target cell dependent manner and the decrease of CD107a expression on cell surface of CD8^+^ T cells further confirmed the negative impact of G-CSF on T-cell functionality (Figure 4, Figure S7 in File S1). Preliminary results from multiple inflammatory cytokine profiling of *in vitro* G-CSF treated PBMCs were not conclusive (data not shown). In order to gain insight into the underlying mechanism in G-CSF-induced impairment of T-cell function, subpopulations of T cells should be investigated separately in a larger cohort.

Recently, Samuel et al. described the successful isolation of functional CMV-specific T cells from G-CSF-mobilized donors after short-term stimulation using CMVpp65 overlapping peptide pool [25]. These results are in concordance with our findings, as they also indicate that G-CSF mobilization impairs IFN-γ secretion at the single-cell level, especially in the CD8 subset, resulting in reduced detection and isolation efficiency of antigen-specific T cells as evidenced by IFN-γ secretion assay. Prior to performing expansion and functional assays, Samuel's group preselected the antigen-specific T cells via expression of activation markers such as CD25 or CD154. As the CD25-enriched cells comprised an increased percentage of FoxP3+ T cells, the authors assumed that this product contained a high proportion of Tregs. Ukena *et al.* [27] showed that G-CSF-mobilized Tregs maintain their phenotypic and functional properties, indicating that co-infusion of these cells might result in the suppression of antigen-specific T-cell proliferation. As expected, CD154^+^ G-CSF-mobilized T cells were functionally similar to G-CSF-untreated CD154^+^ T cells, making this isolation strategy more suitable. However, this finding is based on the comparison of a small number of mobilized and non-mobilized donors and not on a comparison of the same donors before and during G-CSF treatment, as done in this study.

### Implications for scheduling T-cell donations for adoptive transfer

While avoiding additional apheresis by using surplus material from mobilized donors might be beneficial for the donor, it remains unclear whether the G-CSF-treated T cells will achieve control of viral reactivation *in vivo*. Furthermore, seropositive patients with grafts from seronegative donors were the group at highest risk of (recurrent) CMV reactivation [18]. The G-CSF-induced impairment of T-cell function observed here might also affect the potential for generating antigen-specific T cells from those naive donors. We and others have suggested and explored the possibility of banking antiviral CTLs from seropositive donors [52] to provide third-party antiviral CTLs for broader applicability [31]. In addition, G-CSF was shown to induce reactivation of CMV in hematopoietic cells in a humanized mouse model [53]. Thus, isolation of CTL for adoptive transfer before G-CSF mobilization or after the effects of G-CSF have completely worn off might be preferable. Therefore, the duration of the G-CSF effects has to be determined.

### Period of influence of G-CSF on T-cell function

The duration and characteristics of functional impairment of T-cell functions after G-CSF treatment were investigated in several studies. Toh and colleagues [23] showed that granzyme B is still dysregulated three weeks after G-CSF treatment. The Hernandez study [24], which analyzed samples between two and six months after treatment, showed that G-CSF induces high but temporal gene deregulation. Overall, it is not exactly clear how long the effect of G-CSF on T cells, especially antigen-specific T cells, lasts. Gene expression studies of antigen-specific donor T cells before and at defined time points after G-CSF mobilization could answer this question. However, such a study is complicated by the recruitment of high numbers of unrelated HSC donors, who often live far away from the transplant center and thus are not available for further analyses.

Apart from considerations for adoptive T-cell transfer, the duration of G-CSF influence in each donor, in particular the effects on T-cell function after HSC donation should be monitored to determine when T cells recover. This might be essential for donor safety. There are reports showing that donors suffer from side effects like bone pain, headache and flu-like symptoms [54], but there are no studies assessing the donor's risk of infections after G-CSF treatment and HSC donation. However, one should consider that the donors' immune system might be weakened by impaired T-cell function for a prolonged time period rendering them more susceptible to infections.

### Conclusions

Our results demonstrate that antiviral T-cell function is impaired after *in vivo* G-CSF treatment. This suggests that cells of G-CSF-treated donors might not be the best choice in cases where donor lymphocyte infusion or adoptive T-cell transfer is required for treatment of viral reactivation early after transplantation. Further studies are needed to determine whether the adoptive transfer of G-CSF-mobilized T cells is comparable to that of cells from non-mobilized donors or mobilized donors in whom the effects of G-CSF have worn off. In order to increase donor safety, the possibility of higher susceptibility to infections after G-CSF treatment should be investigated and the time needed for T-cell recovery should be determined.

## Supporting Information

File S1 **Supporting Information files.** **Figure S1 in File S1. Effects of sample storage time and temperature.** Multimer analysis is usually performed on whole blood samples stored at room temperature within 24 h. As apheresis and graft processing took longer, some samples were analyzed later. Thus, we evaluated the influence of storage time and temperature on the reliability of the results. Nine patient samples were analyzed consecutively to detect changes in CD3, mNeg (non-specific background) and total percentages of mCMV_pp65_A02 and/or mCMV_pp65_B07 over time. Representative examples are shown. Storage time is plotted on the X-axis, and percentages of total CD3 (A), mCMV_pp65_A02 (B) and mCMV_pp65_B07 (C) on the Y-axis. Staining results for fresh samples (unstored) are depicted as a reference line (baseline; dotted horizontal line), and those for samples stored at room temperature (RT) and 4°C as filled circles (black line) and white circles (dashed line), respectively. Changes in CD3 and specific multimer percentages over time were less pronounced at 4°C in all sample types. Background staining did not change significantly at 24 h or 48 h (data not shown). After 48 h of storage at 4°C, a mean of 93% (mCMV_pp65_A02, n = 4) and 96% (mCMV_pp65_B07, n = 5) of the frequency in the fresh sample (baseline) was obtained. After 72 h, the variation from baseline was more pronounced, even in samples stored at 4°C. For example, in the 5 samples analyzed with mCMV_pp65_A02, only 82% and 30% (mean) of the multimer-positive population detected in fresh material was detected after 72 h of storage at 4°C or room temperature, respectively (Table S2 in File S1). Therefore, donor samples analyzed after more than 48 h (n = 2) were excluded from further analysis. As storage at 4°C led to less deviation from baseline (percentage change), it is preferable. **Figure S2 in File S1. Gating strategy.** (A) Gating strategy for one-platform quantification of percentage and total numbers of CD3CD8-double positive T cells. From left to right: A gate is set on the fluorescent beads for calculation of absolute cell numbers. Flow Count over time is recorded in order to detect changes in the flow rate. The 3rd plot from the left shows gating on lymphocytes for exclusion of debris and other mononuclear cells. CD4CD8-double-positive cells are excluded. The percentage of CD3^+^CD4^+^ and CD3^+^CD8^+^ T cells is determined in the lymphocytes (beads and CD4^+^CD8^+^ cells excluded) and the absolute number per µL blood can be calculated. For A and G samples, beads were added to confirm constant flow rates and signal intensity. (B) Gating strategy for quantification of multimer-positive T cells. From top to bottom, staining in all 3 sample types is shown. The percentage of multimer positive cells is given as percentage of CD3^+^CD8^+^ T cells. FSc – forward scatter, SSc – side scatter, FITC - fluorescein isothiocyanate, PE - Phycoerythrin, PCy7 – Phycoerythrin-Cyanin 7, WBM – whole blood mobilized, A – material from apharesis filter, G – material from graft quality control, Lymph – lymphocyte gate. For multimer abbreviations, please refer to Table S1 in File S1. **Figure S3 in File S1.** ***In vitro*** **application of G-CSF reduces IFN-γ production in PBMCs.** The optimal G-CSF concentration for stimulation was determined by treating PBMCs with different concentrations (5 to 50 ng/ml) of G-CSF for one week and subsequent stimulation with anti-CD3 monoclonal antibodies on day 7. IFN-γ production was measured by intracellular staining and flow cytometry. Results of four independent experiments are shown and expressed as mean ± SD. Asterisks indicate significant differences (**p<0.01). The IFN-γ production in untreated PBMCs (controls) was used as the reference value (100%). The percentage of IFN-γ-expressing cells decreased after G-CSF. A strong effect was achieved using 10 ng/ml G-CSF. At this dose, only 38.73% of IFN-γ-producing cells were retrieved on day 7. Because there was no significant difference after treatment with higher concentrations of G-CSF, 10 ng/ml G-CSF was used in further experiments. **Figure S4 in File S1. Staining with specific multimers is not significantly influenced by sample type.** (A) Unspecific background staining (mNeg): The background detected (mNeg) is shown for each donor sample and the sample type is indicated on the X-axis. Groups were compared by Kruskal-Wallis analysis followed by Dunn's multiple comparison. (B) Exemplary analysis of mCMV_pp65_A02 staining in different samples (Kruskal-Wallis test followed by Dunn's multiple comparison) in CMV-seropositive donors. (C–E) Blots C to E show the comparison of detection of multimer positive cells in the mobilized samples (WBM, A, G) to freshly isolated whole blood (WB). Percentages detected in the mobilized samples only differed slightly from those in WB samples. Sample sources: (A) apheresis tubing set, (G) graft, (WB) whole blood, (WBM) mobilized whole blood on day of apheresis. **Figure S5 in File S1. Multimer staining examples.** Examples for multimer staining in the different sample types (WBM – whole blood mobilized, A – material from apharesis filter, G – material from graft quality control) in 5 donors are depicted. The total sum of multimer positive CD3^+^CD8^+^ T cells after substraction of background (mNeg) for CMV (ΣmCMV) and EBV (ΣmEBV) is given. These values are also reported in Figure 1A and 1B. Plots show CD8-FITC signal versus multimer-PE signal gated on CD3^+^CD8^+^ T cells. **Figure S6 in File S1. Multimer staining with mCMV_pp65_A02 (tetramer) and mCMV_IE1_A02 (pentamer).** Staining examples in CMV-seropositive (CMV^+^, upper row) and CMV-seronegative (CMV-, lower row) donors are given, for each donor, all 3 samples types are shown (WBM – whole blood mobilized, A – material from apharesis filter, G – material from graft quality control). Staining with mCMV_pp65_A02 (tetramer) gives clear populations in CMV^+^ donors and low background in CMV-donors. In contrast, staining with mCMV_IE1_A02 (pentamer) results in less defined populations, furthermore positive staining results are found in CMV-donors (e.g. D#059, D#072, D#101). **Figure S7 in File S1. Functional assays for** ***in vitro*** **G-CSF-treated pCMV_pp65_A02-stimulated cells on day 7.** (A) IFN-γ ELISpot reveals a decrease in IFN-γ secretion after G-CSF treatment. Unstimulated cells served as control. Means of spots per well (spw) are indicated. (B) Granzyme B ELISpot shows a reduction in granzyme B secretion in a target cell dependent manner. Unloaded target cells served as a control. Means of spw are indicated. (C) Representative staining results from the CD107a degranulation assay show a reduced degranulation activity of CD8+ T cells after G-CSF treatment. **Figure S8 in File S1. Follow up in R^+^/D^+^ patients.** (A–C) Number of CMV-CTLs detected in the patient and donor before HSCT and post-HSCT follow-up in patients UPN2104 (A), UPN2131 (B) and UPN2145 (C). CMV reactivation led to expansion of CMV-CTL in UPN2145 (C). **Figure S9 in File S1. Overview of CMV-serostatus of recipients and donors at Hannover Medical School (MHH).** Number of CMV-seropositive patients with seropositive donors (R^+^D^+^; dotted bars) and seronegative donors (R^+^D^−^; striped bars) per year. **Table S1 in File S1. Overview of multimers and antigens used.** **Table S2 in File S1. Variation of total multimer-positive population from baseline (fresh samples) after 24, 48 and 72 hoursof storage at 4°C or room temperature (RT).** **Table S3 in File S1. Donors with no, low and high percentages of multimer-positive T cells.** **Table S4 in File S1. Sequence alignment for the A02-restricted IE-1 epitope VLAELVKQI.**
(DOC)
