# Supplementary material for: Impaired Functionality of Antiviral T Cells in G-CSF Mobilized Stem Cell Donors: Implications for the Selection of CTL Donor
Source: PLoS One. 2013 Dec 4;8(12):e77925. doi: 10.1371/journal.pone.0077925 (PMC3850912; doi:10.1371/journal.pone.0077925)
Supplement: File S1 — Supporting Information files. Figure S1 in File S1. Effects of sample storage time and temperature. Multimer analysis is usually performed on whole blood samples stored at room temperature within 24 h. As apheresis and graft processing took longer, some samples were analyzed later. Thus, we evaluated the influence of storage time and temperature on the reliability of the results. Nine patient samples were analyzed consecutively to detect changes in CD3, mNeg (non-specific background) and total percentages of mCMV_pp65_A02 and/or mCMV_pp65_B07 over time. Representative examples are shown. Storage time is plotted on the X-axis, and percentages of total CD3 (A), mCMV_pp65_A02 (B) and mCMV_pp65_B07 (C) on the Y-axis. Staining results for fresh samples (unstored) are depicted as a reference line (baseline; dotted horizontal line), and those for samples stored at room temperature (RT) and 4°C as filled circles (black line) and white circles (dashed line), respectively. Changes in CD3 and specific multimer percentages over time were less pronounced at 4°C in all sample types. Background staining did not change significantly at 24 h or 48 h (data not shown). After 48 h of storage at 4°C, a mean of 93% (mCMV_pp65_A02, n = 4) and 96% (mCMV_pp65_B07, n = 5) of the frequency in the fresh sample (baseline) was obtained. After 72 h, the variation from baseline was more pronounced, even in samples stored at 4°C. For example, in the 5 samples analyzed with mCMV_pp65_A02, only 82% and 30% (mean) of the multimer-positive population detected in fresh material was detected after 72 h of storage at 4°C or room temperature, respectively (Table S2 in File S1). Therefore, donor samples analyzed after more than 48 h (n = 2) were excluded from further analysis. As storage at 4°C led to less deviation from baseline (percentage change), it is preferable. Figure S2 in File S1. Gating strategy. (A) Gating strategy for one-platform quantification of percentage and total numbers of CD3CD8-double p [file pone.0077925.s001.doc]

**Supplementary Material**

**Supplementary Figures**

**Figure S1. Effects of sample storage time and temperature**

**
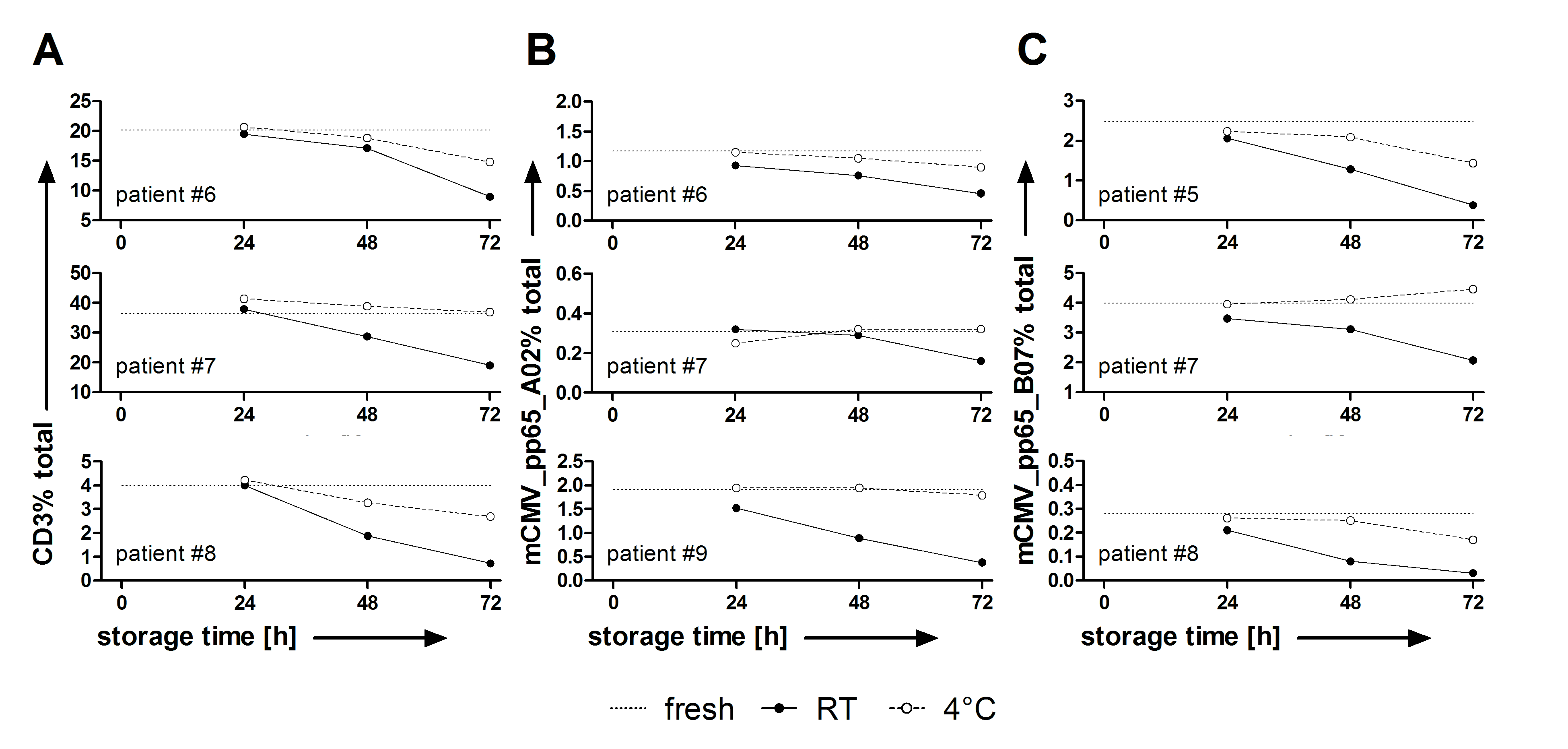
**

**Figure S2. Gating strategy**

| 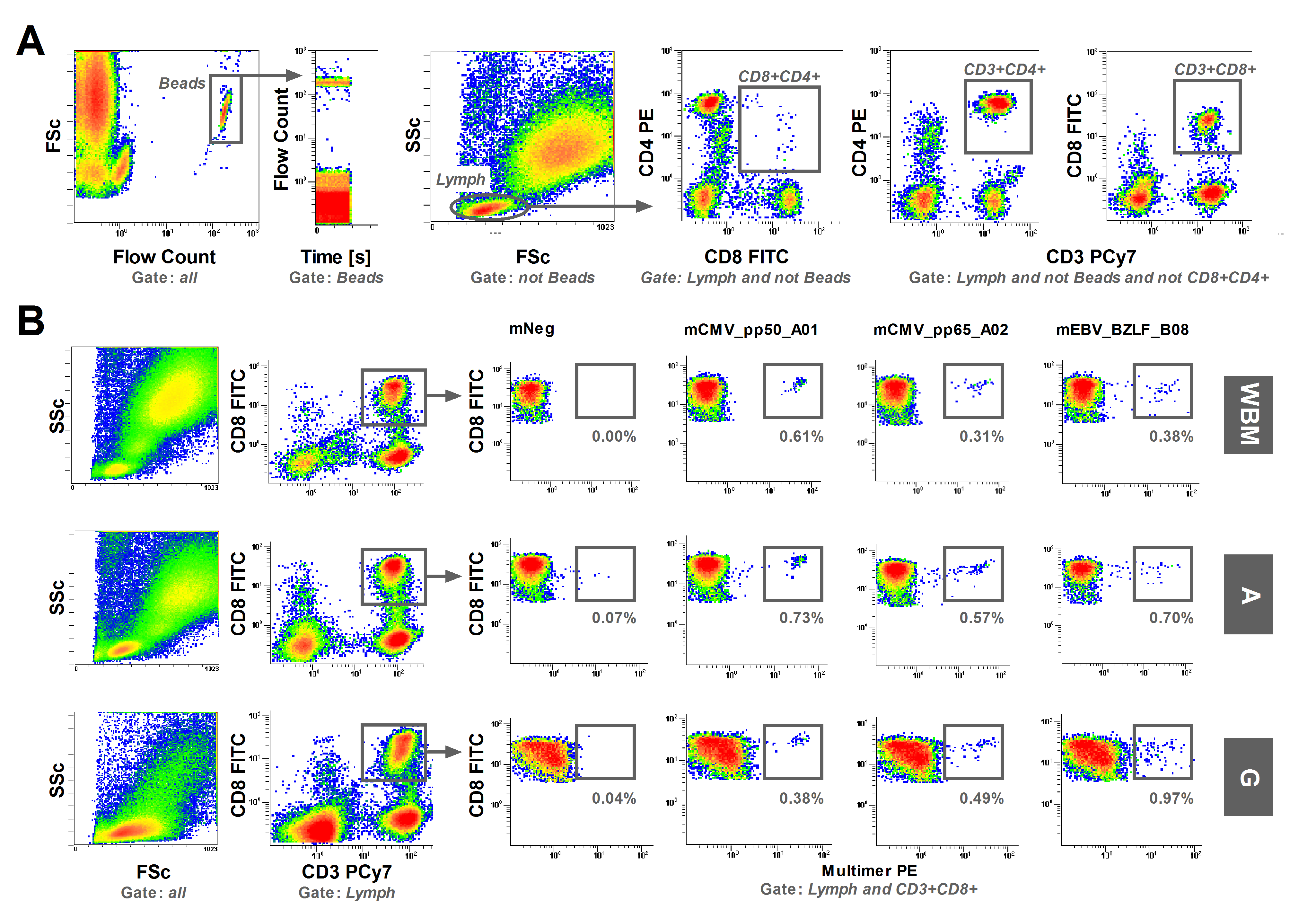 |
| --- |

**Figure S3. *In vitro* application of G-CSF reduces IFN-**γ **production in PBMCs
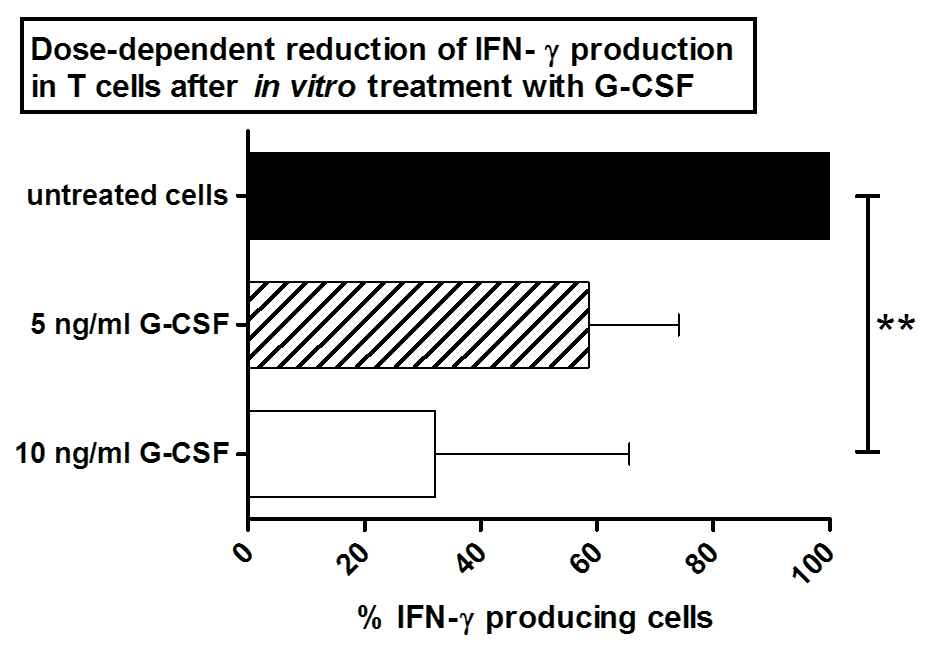
**

**Figure S4. Staining with specific multimers is not significantly influenced by sample type**

##
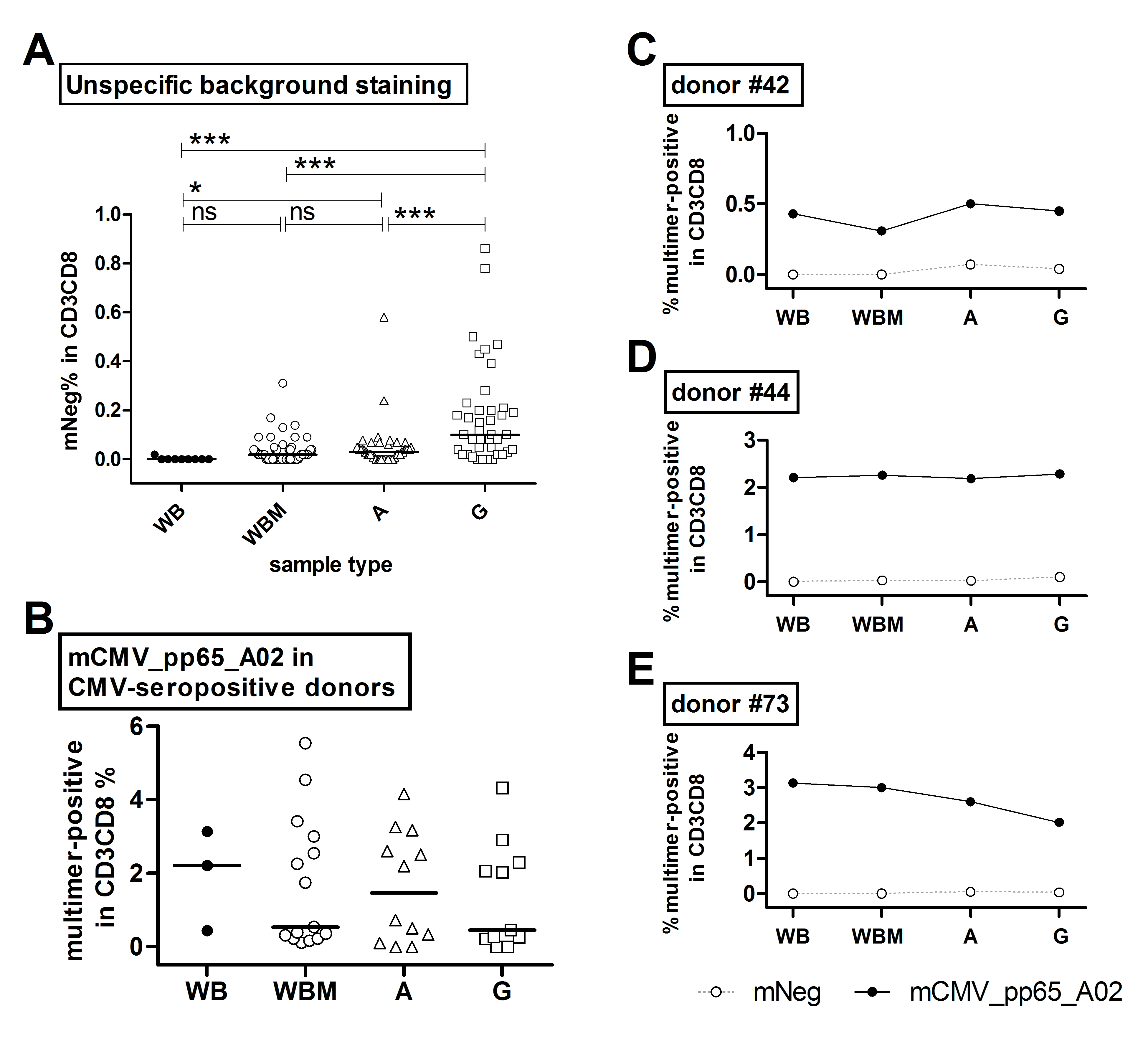


**Figure S5. Multimer staining examples**

| 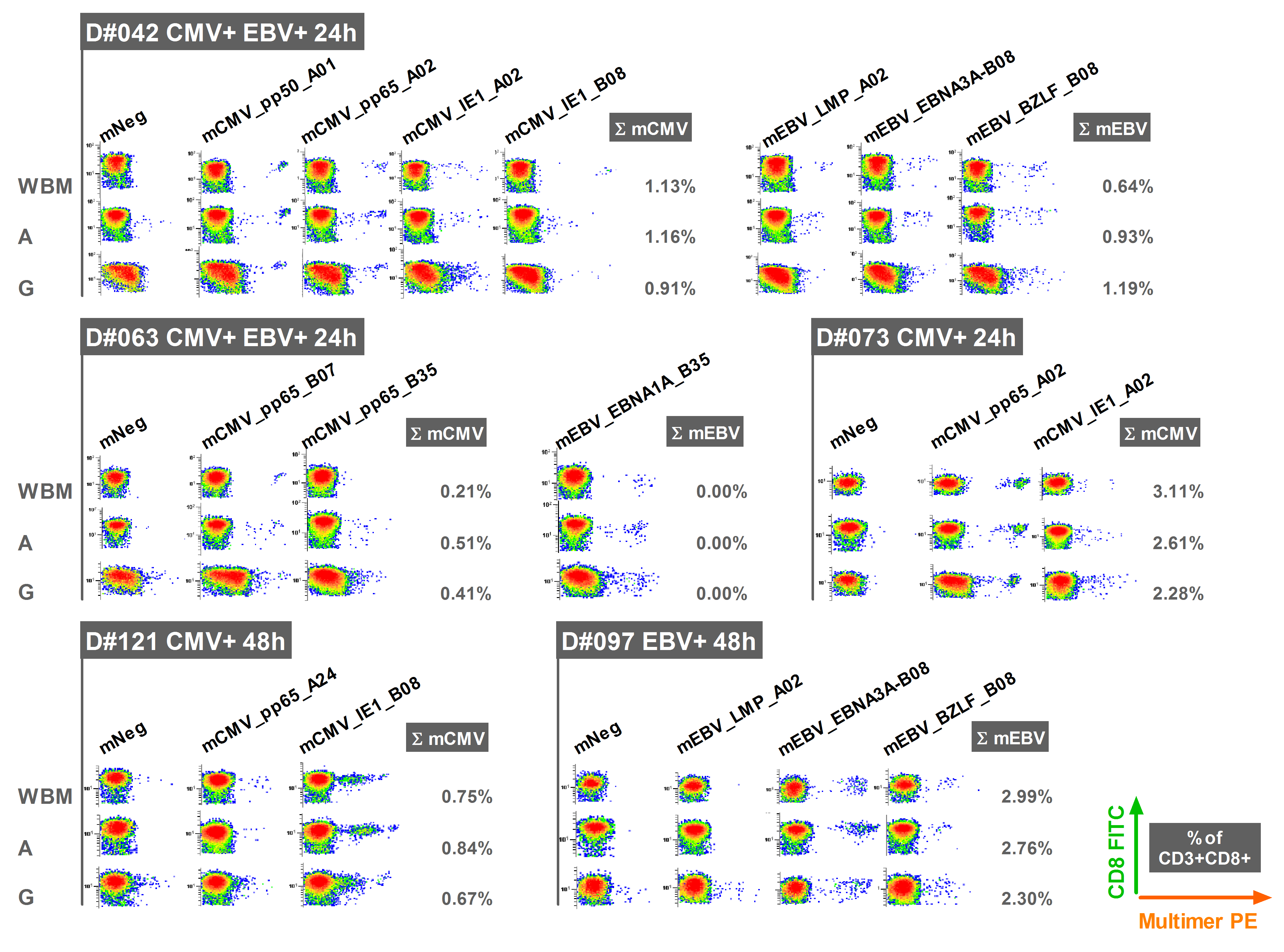 |
| --- |

**Figure S6. Multimer staining with mCMV_pp65_A02 (tetramer) and mCMV_IE1_A02 (pentamer)**

| 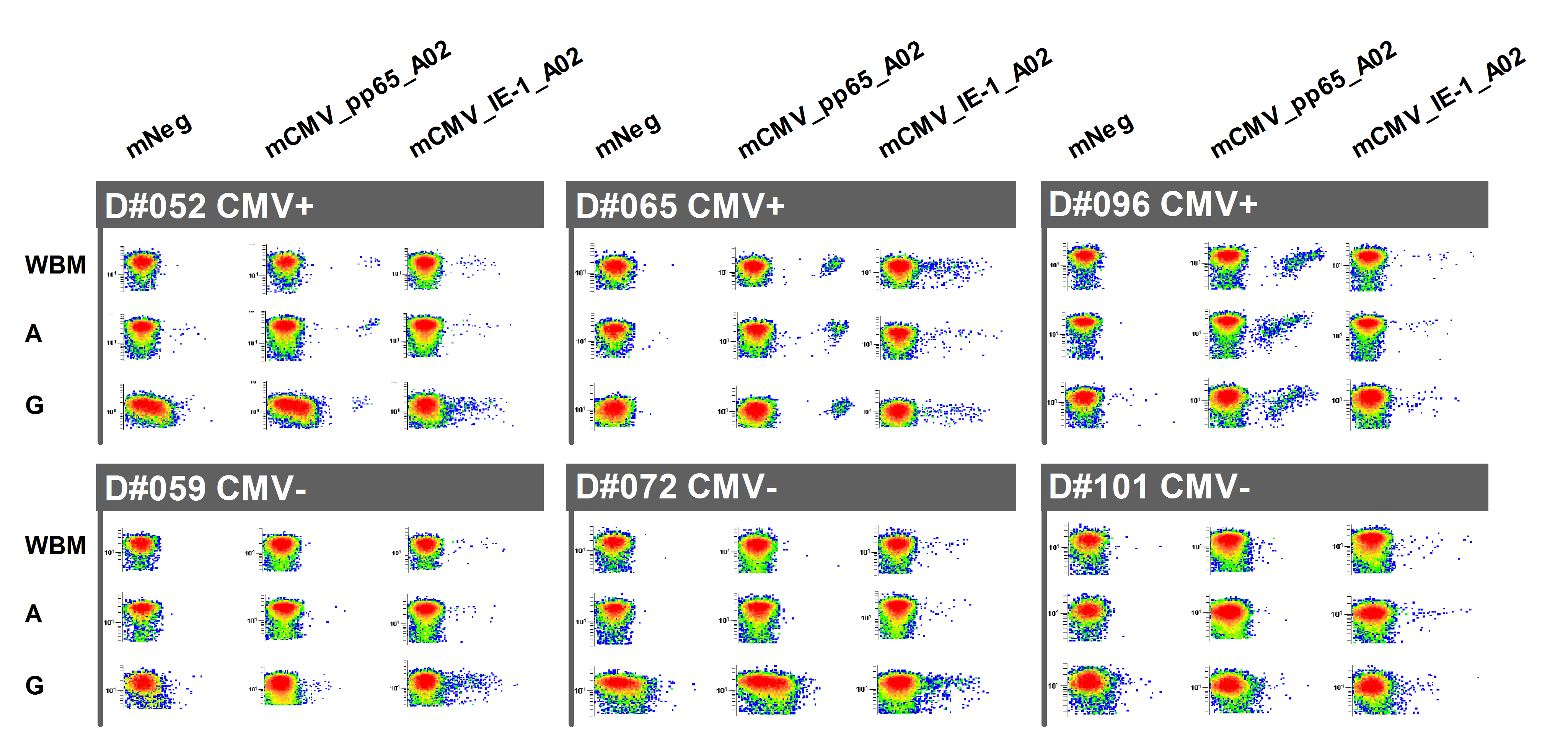 |
| --- |

**Figure S7. Functional assays for *in vitro* G-CSF*-*treated pCMV_pp65_A02-stimulated cells on day 7**


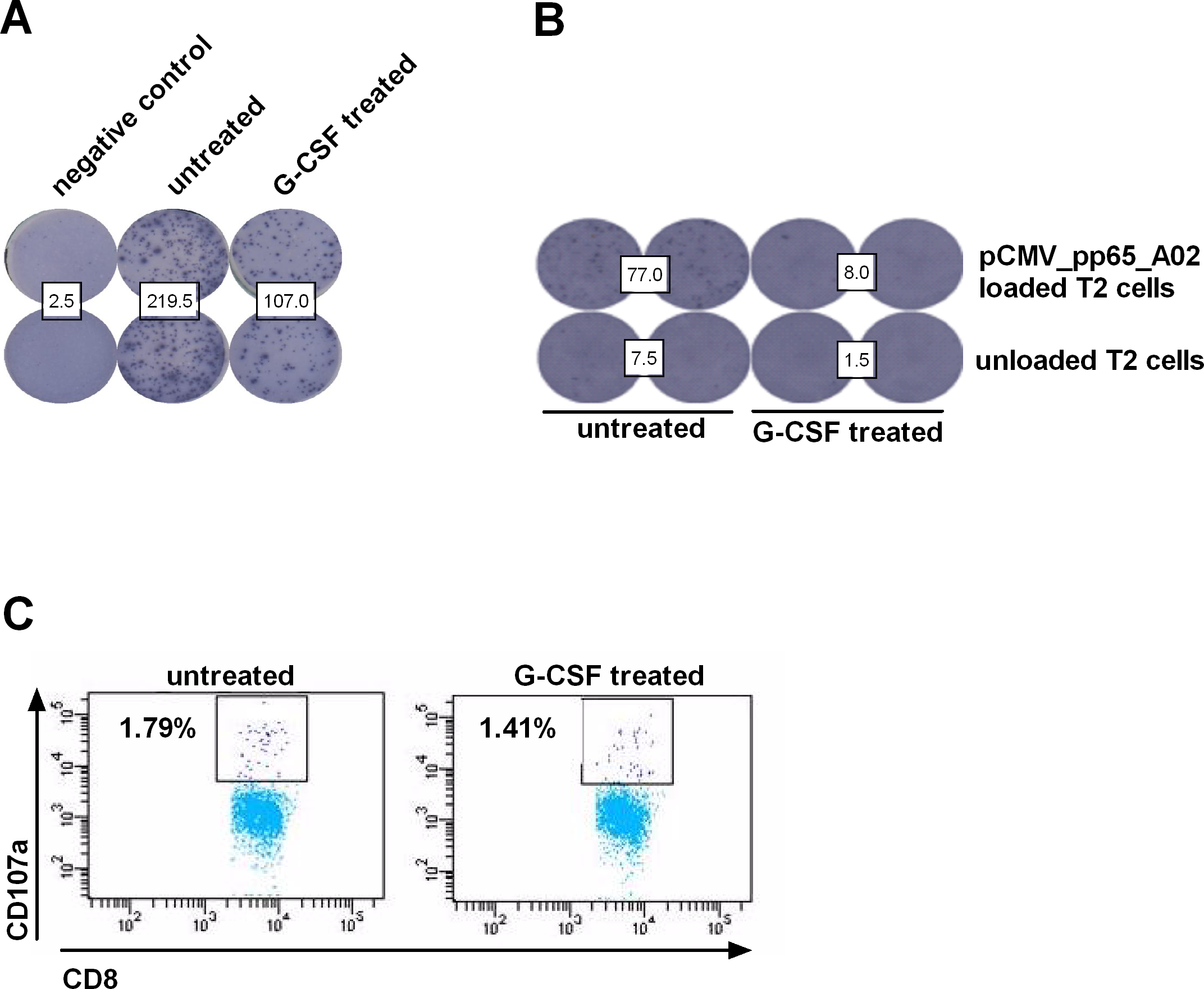


**Figure S8. Follow up in R^+^/D^+^ patients** **
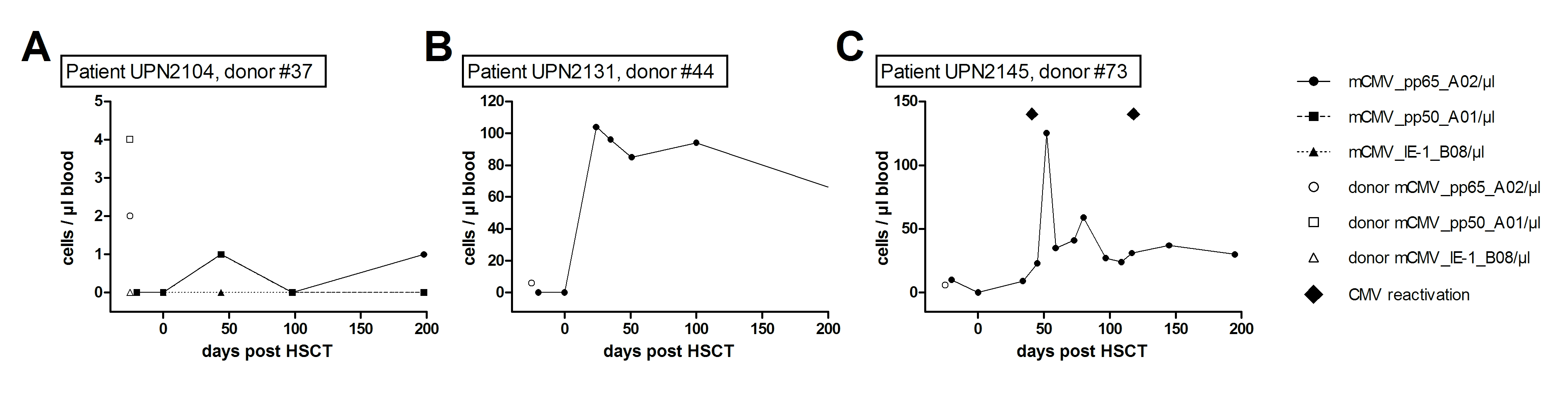
**

**Figure S9. Overview of CMV-serostatus of recipients and donors at Hannover Medical School (MHH)**

**
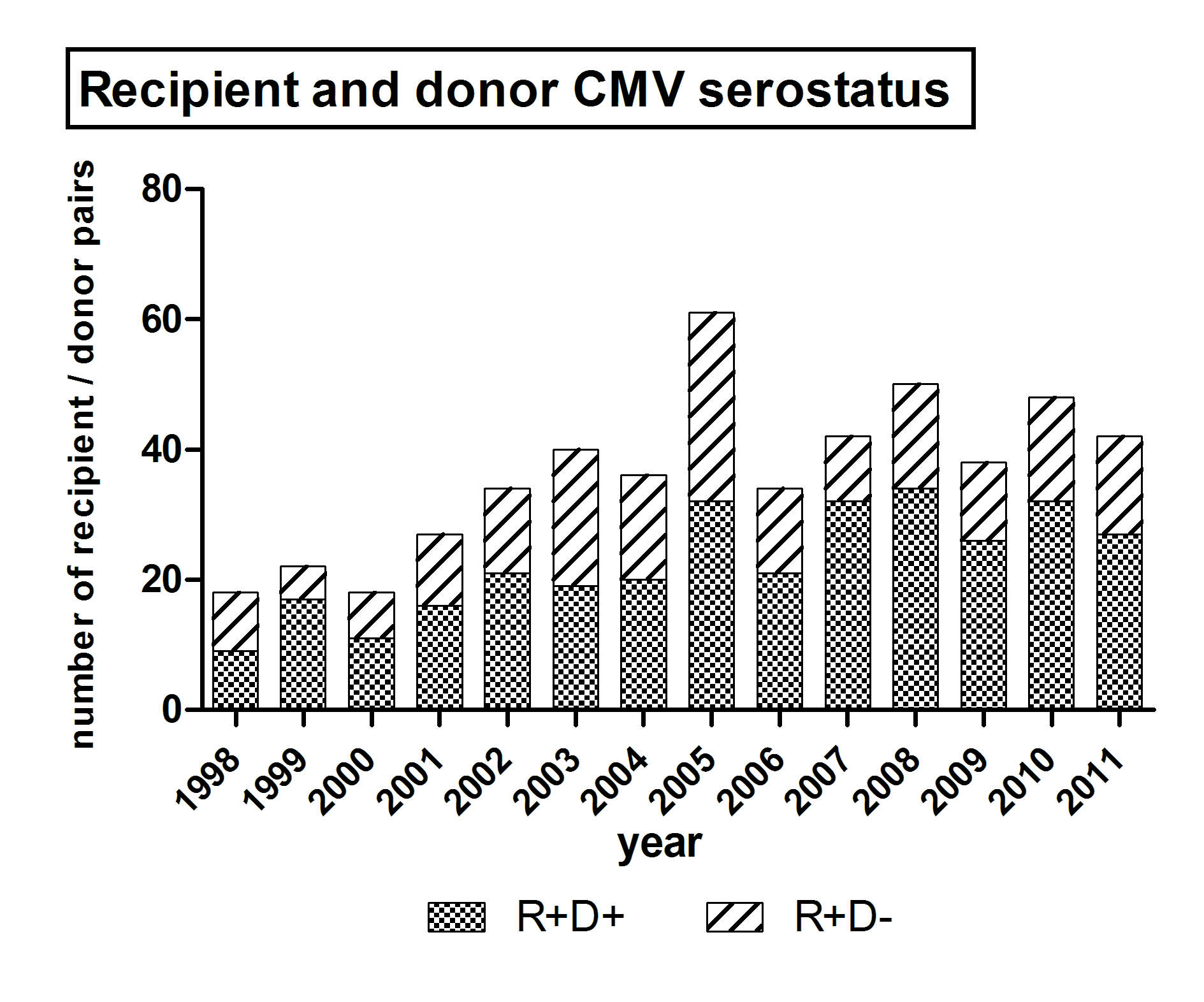
**

**Supplementary Tables**

**Table S**1. Overview of multimers and antigens used

| **Reagent type** | **Experiment** | **Provider** | **HLA-molecule** | **Organism (virus), source protein** | **Peptide sequence** | **Conjugated dye** | **Abbreviation (reagent_virus_source protein_HLA-moleculePeptide)** |
| --- | --- | --- | --- | --- | --- | --- | --- |
| Tetramer | Multimer staining | Beckman Coulter | mix | n/a | n/a (nonsense peptide) | PE | mNeg |
| Tetramer | Multimer staining | Beckman Coulter | A*01:01 | CMV, pp50 | VTEHDTLLY | PE | mCMV_pp50_A01 |
| Tetramer | Multimer staining | Beckman Coulter | A*02:01 | CMV, pp65 | NLVPMVATV | PE | mCMV_pp65_A02 |
| Tetramer | Multimer staining | Beckman Coulter | A*24:02 | CMV, pp65 | QYDPVAALF | PE | mCMV_pp65_A24 |
| Tetramer | Multimer staining | Beckman Coulter | B*07:02 | CMV, pp65 | TPRVTGGGAM | PE | mCMV_pp65_B07 |
| Tetramer | Multimer staining | Beckman Coulter | B*08:01 | CMV, IE-1 | ELRRKMMYM | PE | mCMV_IE-1_B08 |
| Tetramer | Multimer staining | Beckman Coulter | B*35:01 | CMV, pp65 | IPSINVHHY | PE | mCMV_pp65_B35 |
| Tetramer | Multimer staining | Beckman Coulter | A*02:01 | EBV, BMLF1 | GLCTLVAML | PE | mEBV_BMLF-1_A02 |
| Pentamer | Multimer staining | Proimmune | A*02:01 | CMV, pp65 | NLVPMVATV | PE | mCMV_pp65_A02 |
| Pentamer | Multimer staining | Proimmune | A*02:01 | CMV, IE1 | VLAELVKQI | PE | mCMV_IE-1_A02 |
| Pentamer | Multimer staining | Proimmune | B*35:01 | CMV, pp65 | IPSINVHHY | PE | mCMV_pp65_B35 |
| Pentamer | Multimer staining | Proimmune | B*08:01 | EBV, BZLF | RAKFKQLL | PE | mEBV_BZLF_B08 |
| Pentamer | Multimer staining | Proimmune | B*08:01 | EBV, EBNA3A | FLRGRAYGL | PE | mEBV_EBNA3A_B08 |
| Pentamer | Multimer staining | Proimmune | B*35:01 | EBV, EBNA1 | HPVGEADYFEY | PE | mEBV_EBNA1_B35 |
| Pentamer | Multimer staining | Proimmune | A*01:01 | ADV, Hexon | TDLGQNLLY | PE | mADV_Hexon_A01 |
| Pentamer | Multimer staining | Proimmune | A*24:02 | ADV, Hexon | TYFSLNNKF | PE | mADV_Hexon_A24 |
| Pentamer | Multimer staining | Proimmune | B*07:02 | ADV, Hexon | KPYSGTAYNAL | PE | mADV_Hexon_B07 |
| Peptide pool | ELISpot | Miltenyi Biotec | n/a | pp65 | overlapping 15-mers | n/a | ppCMV_pp65 |
| Peptide pool | ELISpot | Miltenyi Biotec | n/a | IE-1 | overlapping 15-mers | n/a | ppCMV_IE-1 |
| Peptide pool | ELISpot | Miltenyi Biotec | n/a | LMP | overlapping 15-mers | n/a | ppEBV_LMP |
| Peptide pool | ELISpot | Miltenyi Biotec | n/a | EBNA-1 | overlapping 15-mers | n/a | ppEBV_EBNA |
| Peptide pool | ELISpot | Miltenyi Biotec | n/a | BZLF | overlapping 15-mers | n/a | ppEBV_BZLF |
| Peptide pool | ELISpot | Miltenyi Biotec | n/a | Hexon | overlapping 15-mers | n/a | ppADV_Hexon |
| Single peptide | T-cell stimulation/ELISpot | Proimmune | A*02:01 | CMV, pp65 | NLVPMVATV | n/a | pCMV_pp65_A02 |
| Single peptide | T-cell stimulation/ELISpot | Proimmune | B*35:01 | CMV, pp65 | IPSINVHHY | n/a | pCMV_pp65_B35 |
| Single peptide | T-cell stimulation/ELISpot | Proimmune | B*08:01 | EBV, BZLF | RAKFKQLL | n/a | pEBV_BZLF_B08 |
| Single peptide | T-cell stimulation/ELISpot | Proimmune | B*35:01 | EBV, EBNA1 | HPVGEADYFEY | n/a | pEBV_EBNA1_B35 |

**Table S**2. Variation of total multimer-positive population from baseline (fresh samples) after 24, 48 and 72 hours of storage at 4°C or room temperature (RT)

| **Multimer** | **Samples** | **Fresh (100%)** | **Mean % relative to baseline** | | | | | |
| --- | --- | --- | --- | --- | --- | --- | --- | --- |
|  |  |  | **24 h / 4°C** | **24 h / RT** | **48 h / 4°C** | **48 h / RT** | **72 h / 4°C** | **72 h / RT** |
| **mCMV-pp65-A02** | 5 | 0,84% | 103% | 104% | 93% | 57% | 82% | 30% |
| **mCMV-pp65-B07** | 4 | 2,07% | 88% | 77% | 96% | 68% | 90% | 37% |

**Table S3. Donors with no, low and high percentages of multimer-positive T cells**

|  |  |  | **MEAN*** | **MEDIAN*** | **No (<0.05%)** | **Low (0.05-0.30%)** | **High (>0.3%)** | **Number of donors tested** |
| --- | --- | --- | --- | --- | --- | --- | --- | --- |
| **CMV** | **mCMV_pp50_A01** | **all** | 0,43% | 0,45% | 4 | 0 | 7 | 11 |
|  |  | **seropositive** | 0,67% | 0,52% | 0 | 0 | 7 | 7 |
|  |  | **seronegative** | 0,01% | 0,00% | 4 | 0 | 0 | 4 |
|  | **mCMV_pp65_A02** | **all** | 0,73% | 0,05% | 15 | 6 | 10 | 31 |
|  |  | **seropositive** | 1,48% | 0,53% | 0 | 5 | 10 | 15 |
|  |  | **seronegative** | 0,02% | 0,02% | 15 | 1 | 0 | 16 |
|  | **mCMV_IE1_A02** | **all** | 0,62% | 0,17% | 3 | 8 | 9 | 20 |
|  |  | **seropositive** | 0,83% | 0,13% | 1 | 6 | 4 | 11 |
|  |  | **seronegative** | 0,35% | 0,35% | 2 | 2 | 5 | 9 |
|  | **mCMV_pp65_B07** | **all** | 0,83% | 0,04% | 6 | 2 | 4 | 12 |
|  |  | **seropositive** | 2,46% | 2,64% | 0 | 0 | 4 | 4 |
|  |  | **seronegative** | 0,02% | 0,01% | 6 | 2 | 0 | 8 |
|  | **mCMV_pp65_A24** | **all** | 0,02% | 0,01% | 14 | 2 | 0 | 16 |
|  |  | **seropositive** | 0,02% | 0,01% | 8 | 1 | 0 | 9 |
|  |  | **seronegative** | 0,02% | 0,01% | 6 | 1 | 0 | 7 |
|  | **mCMV_IE1_B08** | **all** | 0,68% | 0,08% | 2 | 3 | 2 | 7 |
|  |  | **seropositive** | 0,95% | 0,08% | 0 | 3 | 2 | 5 |
|  |  | **seronegative** | 0,01% | 0,01% | 2 | 0 | 0 | 2 |
|  | **mCMV_pp65_B35** | **all** | 0,10% | 0,03% | 7 | 3 | 1 | 11 |
|  |  | **seropositive** | 0,13% | 0,04% | 5 | 2 | 1 | 8 |
|  |  | **seronegative** | 0,04% | 0,02% | 2 | 1 | 0 | 3 |
| **EBV** | **mEBV_LMP2_A02** | **all** | 0,16% | 0,04% | 16 | 10 | 3 | 29 |
|  |  | **seropositive** | 0,16% | 0,04% | 15 | 10 | 3 | 28 |
|  |  | **seronegative** | n/a | n/a | 1 | 0 | 0 | 1 |
|  | **mEBV_EBNA3A_B08** | **all** | 0,71% | 0,20% | 0 | 3 | 2 | 5 |
|  |  | **seropositive** | 0,71% | 0,20% | 0 | 3 | 2 | 5 |
|  |  | **seronegative** | n/a | n/a | n/a | n/a | n/a | 0 |
|  | **mEBV_BZLF_B08** | **all** | 0,51% | 0,51% | 0 | 1 | 4 | 5 |
|  |  | **seropositive** | 0,51% | 0,51% | 0 | 1 | 4 | 5 |
|  |  | **seronegative** | n/a | n/a | n/a | n/a | n/a | 0 |
|  | **mEBV_EBNA1_B35** | **all** | 0,27% | 0,11% | 3 | 2 | 3 | 8 |
|  |  | **seropositive** | 0,30% | 0,17% | 2 | 2 | 3 | 7 |
|  |  | **seronegative** | n/a | n/a | 1 | 0 | 0 | 1 |
| **ADV**** | **mADV_Hexon_A01** | **all** | 0,23% | 0,04% | 8 | 3 | 1 | 12 |
|  | **mADV_Hexon_B07** | **all** | 0,36% | 0,26% | 2 | 4 | 6 | 12 |
|  | **mADV_Hexon_A24** | **all** | 0,15% | 0,14% | 2 | 8 | 1 | 11 |

* Mean values calculated from 1-4 samples per patient. ** for ADV, no grouping according to serostatus was possible

**Table S4. Sequence alignment for the A02-restricted IE-1 epitope VLAELVKQI**

| **Source** | **Amino acid (aa) position** | | | | | | | | | | | **Overlap** |
| --- | --- | --- | --- | --- | --- | --- | --- | --- | --- | --- | --- | --- |
|  | start | 1 | **2** | 3 | 4 | 5 | **6** | 7 | 8 | **9** | stop |  |
| HLA-A*02:01_epitope_motif | 1 |  | **L** |  |  |  | **V** |  |  | **V** | 9 |  |
| pCMV-IE1-A2 | 1 | V | **L** | A | E | L | **V** | K | Q | **I** | 9 |  |
| *Lactobacillus paracasei* | 68 | V | **L** | A | E | L | **V** | K | **D** | **I** | 76 | 8 of 9, #8 mismatched |
| *Burkholderia sp.* | 273 | V | **L** | A | E | L | **V** | K | Q |  | 280 | 8 of 9, #9 mismatched |
| *Coniophora puteana* | 61 | V | **L** | A | E | L | **V** | K | **N** | **I** | 069 | 8 of 9, #8 mismatched |
| *Rhizopus delemar* | 276 | V | **V** | A | E | L | **V** | K | Q | **I** | 284 | 8 of 9, #2 mismatched |
| *Enterobacteria phage RB49* | 196 | V | **L** | A | E | L | **V** | K | Q |  | 203 | 8 of 9, #9 mismatched |

Similar sequences can be found in ubiquitous pathogens. Almost all of the sequence fragments carry the anchor amino acid (aa) for HLA-A*02:01 epitopes in position 2 (L) and the auxiliary anchor aa at position 6 (V) (motif search and epitope binding prediction available at SYTPEITHI; http://www.syfpeithi.de/index.html).
